# Transcriptome Analysis of Short-Day Photoperiod Inducement in Adzuki Bean (*Vigna angularis* L.) Based on RNA-Seq

**DOI:** 10.3389/fpls.2022.893245

**Published:** 2022-06-30

**Authors:** Weixin Dong, Dongxiao Li, Lei Zhang, Baozhong Yin, Yuechen Zhang

**Affiliations:** ^1^State Key Laboratory of North China Crop Improvement and Regulation/Key Laboratory of Crop Growth Regulation of Hebei Province/College of Agronomy, Hebei Agricultural University, Baoding, China; ^2^Hebei Open University, Shijiazhuang, China

**Keywords:** adzuki bean, short-day photoperiod inducement, high-throughput sequencing, transcriptomic analysis, qRT-PCR

## Abstract

The flowering characteristics of adzuki bean are influenced by several environmental factors. Light is an important ecological factor that induces flowering in adzuki bean, but to date, there have been few reports on the transcriptomic features of photoperiodic regulation of adzuki bean flowering. This study is based on RNA sequencing (RNA-seq) techniques to elucidate the expression of light-related regulatory genes under short-day photoperiod inducement of adzuki bean flowering, providing an important theoretical basis for its accelerated breeding. Short-day photoperiod inducement of 10 h was conducted for 5 day, 10 day, and 15 day periods on “Tang shan hong xiao dou” varieties, which are more sensitive to short-day photoperiod conditions than the other varieties. Plants grown under natural light (14.5 h) for 5 days, 10 days, and 15 days were used as controls to compare the progress of flower bud differentiation and flowering characteristics. The topmost unfolded functional leaves were selected for transcriptome sequencing and bioinformatics analysis. The short-day photoperiod inducement promoted flower bud differentiation and advanced flowering time in adzuki bean. Transcriptomic analysis revealed 5,608 differentially expressed genes (DEGs) for the combination of CK-5d vs. SD-5d, CK-10d vs. SD-10d, and CK-15d vs. SD-15d. The three groups of the DEGs were analyzed using the Gene Ontology (GO) and the Kyoto Encyclopedia of Genomes and Genomes (KEGG) databases; the DEGs were associated with flowering, photosystem, and the circadian rhythm and were mainly concentrated in the hormone signaling and metabolism, circadian rhythm, and antenna protein pathways; So, 13 light-related genes across the three pathways were screened for differential and expression characteristics. Through the functional annotations of orthologs, these genes were related to flowering, which were supposed to be good candidate genes in adzuki bean. The findings provide a deep understanding of the molecular mechanisms of adzuki bean flowering in response to short-day photoperiod inducement, which laid a foundation for the functional verification of genes in the next step, and provide an important reference for the molecular breeding of adzuki bean.

## Introduction

Adzuki bean is a grain crop in China with high nutritional and medicinal values and plays an important role in the dietary structure of residents. Its annual export volume is between 50,000 and 80,000 tons (Yin et al., 2019). However, the production of adzuki bean across the country has been limited owing to severe floral bud abscission, low and unstable yield, and restricted cultivation area; these are related to environmental factors during the floral development of adzuki bean (Liu et al., 2015). Light is an important ecological factor that induces flowering in plants, and the flowering time and the flower developmental stages are regulated by the duration of sunlight. This process is closely related to the regulation of flower development, structural changes and related physiological processes, and key genes. A deeper understanding of the molecular mechanisms underlying the photoperiodic flower developmental process could help systematically elucidate the biological mechanisms of photoperiodism in adzuki bean (Song Y. H. et al., 2013; Shrestha et al., 2014).

Plant flowering is jointly regulated by photoperiodic, autonomous, vernalization, and gibberellin pathways. Among them, the photoperiodic pathway is the most important (Pajoro et al., 2014; Kong et al., 2016). The metabolic pathways in photoperiodic flower development and flowering have been extensively studied in different plants. Moreover, the molecular mechanisms underlying the regulation of photoperiodic flowering in *Arabidopsis* have been largely elucidated (Jeong and Clark, 2005; Izawa, 2007). FT protein as florigen can be transported from the leaf to the apical meristem of *Arabidopsis* (Corbesier et al., 2007; Cheng et al., 2018). In the apical meristem, FT protein interacts with FLOWERING LOCUS D (FD), 14-3-3 protein, etc., to form a complex that can promote the expression of downstream flowering genes (Taoka et al., 2011). In soybean, 10 E gene loci *(E1*-*E10)* and a *J* locus related to flowering time have been identified to date, of which *E7* and *E8* loci are involved in inhibiting flowering, but no related genes have been reported (Cober and Voldeng, 2001; Cober et al., 2010; Lin et al., 2021). The other E genes have been studied deeply, e.g., *E1* affects flowering time significantly, inhibits under short-day condition, and shows a bimodal diurnal variation pattern under the long-day condition, indicating its response to photoperiod and its dominant effect induced by long-day length (Xia Z. J. et al., 2012). Furthermore, E1 has been shown to act upstream of four FT-like genes in soybean (*Glyma.16G150700, Glyma.16G044100, Glyma.08G363100*, and *Glyma.18G298900*) and play an important role in promoting soybean flowering as members of the photoperiod gene family (Xia Z. J. et al., 2012; Zhai et al., 2014; Liu et al., 2018). *E2*, a homologous gene of *GI* and *GmGIa*, and *e2*/*e2* genotype induce early flowering of soybean by inducing the expression of *GmFT2a*, a flowering gene homolog (Watanabe et al., 2011). *E3* and *E4* are related to phytochrome A, which is associated with the regulation of flowering in soybean. Further analysis by mapping showed that *GmPhyA3* is a candidate gene at the *E3* locus and *GmphyA2* is encoded by E4 (Watanabe et al., 2009; Liu et al., 2008). *E5*, as a gene that does not work alone, may be generated by accidental outcrossing of pollens that have the E2 allele (Dissanayaka et al., 2016). *E6* is a new allele of the homolog *J* of *ELF3*, the early flowering gene of *Arabidopsis*. The long-juvenile (*LJ*), characterized by delayed flowering and maturation, could increase yield under short-day conditions and expand soybean cultivation in lower latitudes (Fang et al., 2021). In the soybean mature gene E9 locus, the abundance of FT2a is directly related to the change in the soybean flowering time (Zhao et al., 2016). At the end of the Gm08 chromosome, there is a new maturation site E10, where FT4 is the most likely candidate gene by protein interaction and plays a regulatory role in soybean flowering time (Samanfar et al., 2017). *J* is a homolog of *EARLY FLOWERING3* (*ELF3*) in *Arabidopsis thaliana*. The J protein binds to the legume-specific FLOWERING suppressor *E1* and downregulates the transcription of the *E1* promoter to release the T(FT) gene, which is the important flowering site and promotes flowering in a short time (Lu et al., 2017). In addition to the *E* genes, *FT* genes also play important roles in soybean flowering (Banfield et al., 1998; Su et al., 2021); two cognates of FT, *GmFT2a* and *GmFT5a*, upregulated under a short-day photoperiod has the same function as FT in *Arabidopsis thaliana*, but the effect of *GmFT5a* is more significant. High expression of *GmFT2a* and *GmFT5a* has been shown in the double mutations of two phytochrome A (*PHYA*) genes under long-day conditions with flowering times slightly earlier than those of the wild type under short-day photoperiods. This indicated that the expression of *GmFT2a* and *GmFT5a* under long daylength conditions is affected by the photoperiod regulation system mediated by *PHYA*. *GmFT2a* and *GmFT5a* coordinately control flowering and enable soybean to adapt to a wide range of photoperiodic environments (Kong et al., 2010). Similarly, in *C. cajan*, among 13 PEBP (FT) family genes, *CcFT6* and *CcFT8* act as crucial florigen T genes involving flowering regulation. The expression of *CcFT6* is upregulated under short-day conditions, whereas the *CcFT8* transcripts are enhanced under long-day photoperiods to promote the plant flowering process, suggesting that they regulate plant flowering through photoperiod-dependent pathways (Tribhuvan et al., 2020). Transcriptome analysis indicated that Mtvrn2 (VERNALISATION2-LIKEVEFS-box gene) mutants of legume *Medicago truncatula* precociously express FTa1 and other suites of genes including floral homeotic genes. Functional FTa1, an FT-like gene, work as the floral activator and has an elevated expression to regulate early flowering under long-day conditions (Jaudal et al., 2016). In chickpea, SNP loci from the CaCLV3_01 gene (*CLAVATA*) within a major CaqDTF1.1/CaqFN1.1 QTL has been identified and verified to be associated with flowering time (DTF) and flower number (FN) traits (Basu et al., 2019).

In summary, research studies on the screening and the functional analysis of flowering-related key genes in *Arabidopsis*, soybean, *C. cajan, Medicago*, chickpea, etc. have provided insights into photoperiodic responses and flowering mechanisms in leguminous plants. For adzuki bean, most studies on flower development have focused on morphology and phenotypic physiology. Previously, we found that the growth index of adzuki bean is largely determined by different short-day induction. The extended short-day induction duration decreased the chlorophyll, soluble sugar, and protein contents; increased enzyme activity; and altered hormone dynamic balance homeostasis (Dong et al., 2016). Our microscopic observation also found that the differentiation phase of inflorescence primordia in flower buds is promoted in adzuki bean plants after short-day induction (Zhang et al., 2021). According to this, “Tang shan hong xiao dou”, a late-maturing variety sensitive to short-day photoperiod conditions, was used to research functional leaf responses to light and flowering under different short-day induction conditions based on transcriptomic analysis (RNA-Seq). Taking flowering-related genes of soybean as a reference, we attempted to search for flowering responsive genes in the genome annotations of adzuki bean to deepen the understanding of the photoperiodic response and the molecular mechanism of flower development in adzuki bean. This study could help to further understand the flowering mechanism of this plant and provide an important theoretical basis for genetic improvement in the high light efficiency of adzuki bean.

## Materials and Methods

### Experimental Material

“Tang shan hong xiao dou,” a late maturing and photoperiod sensitive variety, was used as the experimental material, which was provided by the Research Institute of Grain and Oil Crops, Academy of Agricultural and Forestry Sciences of Hebei, China. The flowering time and maturity time are earlier of this variety than other regularly cultivated cultivars under short-day photoperiod inducement. The experiment was conducted at the teaching experimental station of Hebei Agricultural University, China (38°38, N, 115°E), during the growth seasons in 2021.

### Short-Day Treatment of Plant Materials

This field experiment was performed at the teaching test base of Hebei Agricultural University. Each plot was designed to be 5 m in length, 1 m in width, and 20 cm in depth. Prior to seed sowing, 400 g compound fertilizer (N-P_2_O_5_-K_2_O=15-15-15) was applied to each plot (5 × 1 m) and then plowed. The topsoil (0-20 cm) had 1.54% organic matter, 0.0987% total nitrogen, 84.97 ppm available N, 65.658 ppm available P, and 184.6 ppm available K. The seeds were sown on 24 June and planted in two rows in each plot with a spacing of 15 × 40 cm. When true leaves flatten under natural light (14.5 h), short-day treatment was started with stainless steel shelves placed on the upper side of the plot together with a covering opaque cloth for shading and fixing additional vents for adjusting temperature, CO_2_ concentration, and relative humidity (Table 1). Then, short-day photoperiod was set to 10-h light/14-h dark. At the first leaf expansion stage, three photoperiod treatments were set up by sheltering the seedlings with an opaque cloth during the daytime. These treatments included natural photoperiod (14.5 h) with 5 days (control check-5d), 10 days (control check-10d), and 15 days (control check-15d), and short-day photoperiod treatments with 5 days (short day-5d), 10 days (short day-10d), and 15 days (short day-15d). After shading, all plants grew to the maturity stage under natural light with spraying 0.4% potassium dihydrogen phosphate. Irrigations were conducted at the first flowering stage and the pod setting stage. The diseases and insect pests were controlled according to the actual occurrence situation. The experimental treatment consisted of 18 plots with random block arrangement. Photoperiod treatment was initiated at 8:00 am and terminated at 6:00 pm with a 10-hour photoperiod duration. Intermediate leaves with the uppermost unfolded three complex leaves were taken at 9:00 am to be mixed from five to six plants after shading for 5 days, 10 days, and 15 days. Each treatment was repeated three times, and there were 18 samples marked as SD-5d-1, SD-5d-2, SD-5d-3, CK-5d-1, CK-5d-2, CK-5d-3; SD-10d-1, SD-10d-2, SD-10d-3, CK-10d-1, CK-10d-2, CK-10d-3; SD-15d-1, SD-15d-2, SD-15d-3, CK-15d-1, CK-15d-2, and CK-15d-3. After that, the samples were quick-frozen in liquid nitrogen and stored at −80°C. One week later, RNAs from the samples were extracted and subjected to sequencing analysis.

**Table 1:** Changes in field microclimate with shading treatment.

	**Illumination intensity (lux)**	**CO_**2**_ concentration (ppm)**	**Relative humidity (%)**	**Temperature (**°**C)**
Atmosphere	57,963.33 ± 1,678.58a	451.53 ± 18.72a	78.82 ± 4.77a	26.59 ± 0.89a
Shading	10.63 ± 3.50b	463.88 ± 20.45a	82.33 ± 1.79a	27.28 ± 1.09a

*Values are means ± S.E. The different small letters in the same column indicate statistical significance at 0.05 level by DMRT*.

### Measurement of Meteorological Factors

During the growth stage, light intensity at 20–30 cm above the canopy of the adzuki bean community in the plot was measured using a TES1332 illuminometer (TES1332, Taiwan, China). An LI-6400 portable photosynthetic apparatus (Li-COR, Lincoln, NE, USA) was used to measure the CO_2_ concentration. A HOBO Pro V2 series instrument (U23-002, USA) was used to measure temperature and humidity, which were recorded automatically every hour. The data were exported after the testing was finished.

### Measurement of Plant Growth Traits

Totally, nine plants with uniform growth in each plot were subjected to measure the plant growth traits after photoperiod treatments. The growth traits assessed included plant height, stem diameter, pinch number, leaf area, and shoot dry weight per plant. The plant height was obtained by measuring the distance (cm) from the cotyledon node to the growing point. The stem diameter of plants was determined by using an electronic vernier caliper (United Precision Machine Precision Measurement Limited Company, Shenzhen, China).

The number of main stem nodes was referred to as the number of first nodes starting from the true leaf. The leaf area was measured using a YMJ-B leaf area meter (from Hangzhou Hui Equipment limited company). The shoot dry weight per plant was measured using the conventional oven drying method.

### Anatomical Observation and Slicing Production of Adzuki Bean Flower Buds

According to the division method of Jin and Wang (1995), flower bud differentiation of adzuki bean was divided into the pre-differentiation stage (PD), the inflorescence primordium differentiation stage (IP), the flower primordium differentiation stage (FP), the sepal primordium differentiation stage (SP), the petal primordium differentiation stage (PP), the stamen and carpel primordium differentiation stage (SCP), and the stamen and carpel structural differentiation stage (SCS). After shading was completed (about 20 days after seedling emergence), the top flower buds were taken for each treatment with 20 flower buds. After making paraffin sections, they were observed under a microscope (BS500/BS500-TR biological microscope). More than 70% of flower buds differentiated into a certain period was judged it has entered this developmental period. Production methods of paraffin sections include sampling, fixation, dehydration and transparent, paraffin-embedded, slicing and patch, and HE staining.

### Investigation and Statistics of Flowering Characteristics

The time period when 50% of plants began to blossom was called the flowering period, when three representative plants were selected per treatment with three replicates and nine plants in total. Early flowering time was recorded, and the flowering promotion rate was calculated. The determination formula is as follows:

Flowering promotion rate (%) = [(Control the seedling emergence period to the flowering period-Different treatment of seedling period to flowering period time)×100]/Control the seedling emergence period to the flowering period.
Early flowering time = Control the seedling emergence period to the flowering period – Different treatment of seedling period to flowering period time.

### RNA Extraction and Library Construction

Total RNA was extracted using a mirVana miRNA Isolation Kit (Ambion) following the manufacturer's protocol. RNA integrity was evaluated using an Agilent 2100 Bioanalyzer (Agilent Technologies, Santa Clara, CA, USA). The samples with an RNA integrity number (RIN) ≥ 7 were subjected to the subsequent analysis. The libraries were constructed using a TruSeq Stranded mRNA LTSample Prep Kit (Illumina, San Diego, CA, USA) in accordance with the manufacturer's instructions. Then, these libraries were sequenced on the Illumina sequencing platform (HiSeqTM 2500 or Illumina HiSeq X Ten), and 125-bp/150-bp paired-end reads were generated.

### Quality Control and Mapping

Raw data (raw reads) were processed using Trimmomatic (Bolger et al., 2014). The reads containing ploy-N and the low-quality reads were removed to obtain the clean reads. Then, the clean reads were mapped to the reference genome using hisat2 (Kim et al., 2015).

### Analysis of Differentially Expressed Transcripts (DEGs)

Analysis of DEGs was performed through transcript-level quantification, and FPKM (Roberts et al., 2011) and read count value of each transcript (protein_coding) were calculated using bowtie2 (Langmead and Salzberg, 2012) and eXpress (Roberts and Pachter, 2013). DEGs (read counts >2) were identified using the DESeq (Anders and Huber, 2012) with functions estimate size factors and nbinomtest. *p* < 0.05 and a fold change >2 or fold change < 0.5 were set as the threshold for significantly differential expression. Differentially expressed genes (DEGs) were selected by comparing all genes expressed in adzuki bean leaves under different short-day photoperiod inducement conditions.

### Functional Annotation Analysis of Differentially Expressed Genes

Differential transcripts with a *p* < 0.05 and a difference fold >2 were selected, GO annotation information using Blast2GO software for all the differential genes were obtained, and GO classification statistics for all the differential genes were used for GO using WEGO software. The GO annotation includes three main branches, namely, biological process, molecular function, and cellular component. Gene sequences were annotated to the Kyoto Encyclopedia of Genes and Genomes (KEGG) database by BLAST software alignment, and then the organism's metabolic network was studied.

### Quantitative Real-Time PCR (qRT-PCR) Analysis

To verify the accuracy of the adzuki bean sequencing results, 13 differentially expressed genes were verified again by qRT-PCR using new leaf samples under the same treatment. The specific steps were as follows: Total RNA was extracted from adzuki bean tissues with trizol reagent. Residual genomic DNA in the RNA samples was removed by RNase-free DNase following the manufacturer's protocol (New England Biolabs). In total, one microgram of DNase-treated total RNA was used to synthesize first-strand cDNA using the First Strand cDNA Synthesis Kit (Thermo Fisher Scientific), and real-time RT-PCR was performed with three technical replicates using SYBR Premix Ex Taq (Takara Biotech) on an ABI Applied Biosystems StepOnePlus machine (Life Technologies). Gene expression was calculated using the 2^−ΔΔCt^ method (Kenneth and Schmittgen, 2001). Primers were designed using Primer 5.0, and the primer sequences are listed in Table 2.

**Table 2:** Primers for qRT-PCR.

**Order number**	**Gene ID**	**Forward primer sequence (5^**′**^ **→** **3**^**′**^)**	**Reverse primer sequence (5^**′**^ **→** **3**^**′**^)**
1	ACTIN	F:CTAAGGCTAATCGTGAGAA	R:CGTAAATAGGAACCGTGT
2	LOC108334289	F:CACAATCACAAAGCAGCATCC	R:TTTAGCGAAGTGGCAAGATGA
3	LOC108345251	F:GCCCCTCCTTTTGTTCTCACT	R:CCTCCCACCTTCCATATTGTT
4	LOC108342301	F:TCCTTGACAAATCCAAAACCC	R:GGAAAAGCGAGGAGAAGAAGA
5	LOC108319999	F:TCAGCAGCATCAACAGGACAA	R:GGTCTCAAAGGGGCTATTCAA
6	LOC108320001	F:ATGTCCCTCCATTAGCCTCAG	R:GAACACCCATAGTTCCCCTCA
7	LOC108328360	F:ATCAAAGATGGAGGAACACCC	R:TCCTGCACAATCTACACTTGC
8	LOC108325858	F:TGTTTCAGTTGTTCCTCCGTG	R:GCGTTGCTTGGCTTTTCTAAT
9	LOC108331524	F:AATGGAAAACGGAACTGAGGA	R:CAGAAGGGTGGAAGTGATGCT
10	LOC108344448	F:TCAAAGATAAGACGCAGCACC	R:TGATTGTTAGGGAGGGAGACG
11	LOC108331766	F:AAAAGGGAGGACCAAGAGCAC	R:TGAGTGGCACAACACCTGAAT
12	LOC108328079	F:AAGAAACGCAGAACTTGACCC	R:GCTTGAATGGCAAAGATGAGG
13	LOC108330684	F:AAGAACCCTGGCAGTGTCAAC	R:ATGCCAACATAGCCAATCTCC
14	LOC108340483	F:AACCAATGCCAACCCTCTTAG	R:CAGTGTCCCATCCATAGTCCC

### Data Analysis

In this experiment, the genome of *Vigna angularis* (URL: https://ftp.ncbi.nlm.nih.gov/genomes/all/GCF/001/190/045/) was used as a reference genome (Kang et al., 2015). A total of 121.09 G clean data were obtained by sequence similarity analysis by searching against the reference transcriptome database using our 18 samples. The effective data of each sample were 6.04–7.2 G. The distribution of Q30 bases reached 93.7–94.64%, and the average GC content was 44.10%. Mapping reads to the reference genome to obtain genome alignment of each sample with alignment rate were 97.09–98.22%. The resulting raw data were quality-filtered using NGS QC Toolkit software to obtain high-quality clean reads. Then, the clean reads were aligned using HISAT2 to the reference genome of species by the genome alignment rate. Gene FPKM expression values were quantified using Cufflinks software. Duncan ANOVA and multiple comparisons were carried out at 0.05 level using SPSS 17.0 software, and images were visualized by BS500/BS500-TR biomicroscopy and PhotoshopCS6 software treatment.

## Results

### Plant Growth

The plant height, stem diameter, internode number, leaf area, and aboveground dry weight of adzuki bean were all inhibited under short-day inducement conditions (Table 3). The longer the short-day induction time, the more the decrease in growth index and the earlier the maturity stage. At the flowering stage, compared with CK, the leaf area decreased significantly under all short-day treatments and plant height decreased significantly under SD-15d. At the podding and seed-filling stages, similar results were observed. All the growth indexes had the largest decrease under SD-15d compared with other treatments.

**Table 3:** Growth of adzuki bean under short-day photoperiod inducement.

**Determination stages**	**Treatment**	**Plant height/cm**	**Stem diameter/cm**	**Main stem nodes number**	**Leaf area/cm^**2**^**	**Shoot dry weight/g**
Flowering	CK	27.67 ± 3.93a	0.58 ± 0.06a	14.00 ± 1.00a	37.24 ± 3.90a	8.90 ± 1.23a
	SD-5d	19.60 ± 1.91b	0.47 ± 0.08b	13.67 ± 2.08a	29.68 ± 6.47a	6.95 ± 3.04ab
	SD-10d	19.13 ± 2.58b	0.45 ± 0.11b	12.67 ± 0.58a	26.13 ± 1.21ab	4.30 ± 1.00b
	SD-15d	18.93 ± 2.89b	0.41 ± 0.03b	12.00 ± 1.73a	22.66 ± 4.80b	4.59 ± 2.18b
Podding	CK	33.80 ± 4.10a	0.68 ± 0.12a	18.00 ± 1.00a	51.40 ± 5.22a	18.46 ± 2.37a
	SD-5d	32.10 ± 5.52a	0.64 ± 0.13a	17.33 ± 2.08ab	48.51 ± 1.75a	17.00 ± 3.53a
	SD-10d	30.10 ± 3.50a	0.60 ± 0.07a	16.00 ± 1.73b	45.71 ± 6.89ab	16.23 ± 2.90a
	SD-15d	28.53 ± 2.84a	0.49 ± 0.03b	15.33 ± 0.58b	42.40 ± 2.03b	9.87 ± 1.11a
Seed-fling	CK	41.20 ± 5.90a	0.74 ± 0.11a	20.33 ± 4.04a	55.69 ± 7.70a	52.24 ± 4.70a
	SD-5d	40.37 ± 2.71a	0.73 ± 0.05a	19.00 ± 1.73a	52.92 ± 3.78a	40.87 ± 3.88ab
	SD-10d	37.37 ± 6.06a	0.68 ± 0.04ab	17.00 ± 1.00ab	51.41 ± 8.22a	35.20 ± 4.86ab
	SD-15d	29.97 ± 2.98b	0.62 ± 0.03b	15.33 ± 1.15b	47.59 ± 5.33a	23.05 ± 3.13b

*Values are means ± S.E. The different small letters in the same column indicate statistical significance at 0.05 level by DMRT*.

### Flower Bud Differentiation Process and Flowering Characteristics

**Figure 1:**
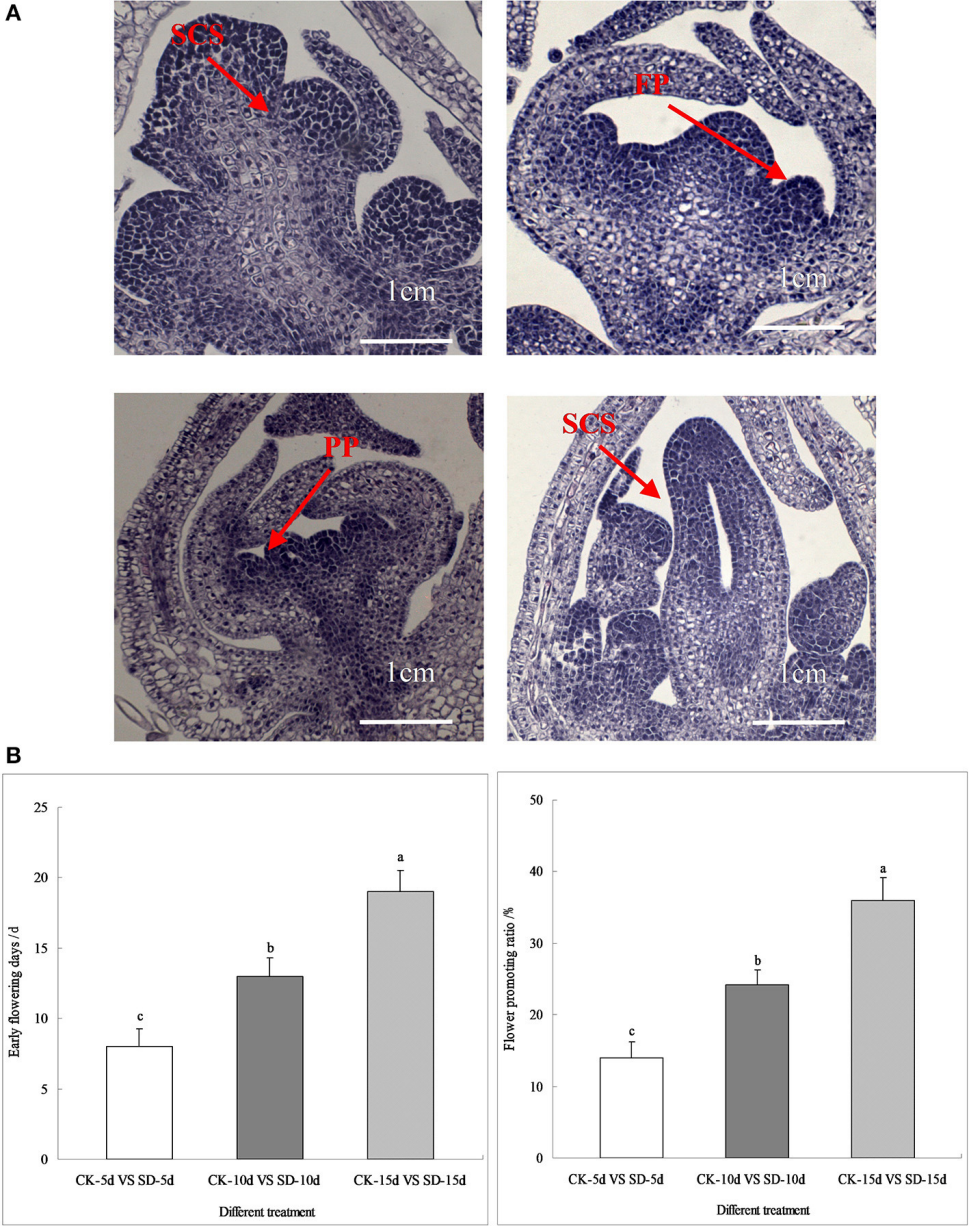
Flower bud differentiation, early flowering days and flower promoting ratio of adzuki bean. **(A)** Pictures of flower bud differentiation processes under different short-day photoperiod treatments. There were three treatments (CK-natural lighting, SD-5d, SD-10d, SD-15d). **(B)** Statistics of early flowering days and flower promoting ratio under different short-day photoperiod treatments (CK-5d vs. SD-5d, CK-10d vs. SD-10d, CK-10d vs. SD-10d). Different letters were represented as Values are means ± S.E. The different lowercase letters above each bar chart indicate statistical significance at 0.05 level by DMRT.

As seen in Figure 1, short-day photoperiod inducement significantly affected the process of floral bud differentiation and the flowering characteristics of adzuki bean. After short-day photoperiod inducement, the differentiation of apical flower buds under different treatments was observed. The findings showed that short-day photoperiod inducement significantly promoted the process of flower bud differentiation in adzuki bean. When the control was at the inflorescence primordium differentiation phase, the apical flower buds of adzuki bean plants under short-day light for 5 days, 10 days, and 15 days had already entered the floral primordium differentiation stage, the petal primordium differentiation stage, and the pistil and stamen differentiation stage (Figure 1A).

Further statistics showed that, compared with the controls, adzuki bean with short-day photoperiod inducement of 5, 10, and 15 days had early flowering induced at 7, 13, and 20 days and a flowering promotion rate of 13.99, 24.19, and 36.02%, respectively. The number of days of induced early flowering and the flowering promotion rate under different short-day photoperiod inducement periods reached significant levels. Comparing CK-15d vs. SD-15d to CK-5d vs. SD-5d and CK-10d vs. SD-10d, the number of days of early flowering was 65.00% and 35.00% earlier, respectively, while the flowering promotion rate was 61.16% and 32.84% earlier, respectively (Figure 1B). It was seen that the late-maturing “Tang shan hong xiao dou” variety was more sensitive to short-day photoperiod conditions, displaying increased reproductive growth by regulating its own vegetative growth that led to earlier flowering by more number of days with a higher flowering promotion rate. This shows that short-day photoperiod inducement promoted flower bud differentiation and the flowering promotion rate of adzuki bean, possibly due to its role in regulating gene expression in flowering metabolic pathways.

### Statistical Analysis and Quality Evaluation of RNA-Seq

The “Tang shan hong xiao dou” variety sampled at 5 days, 10 days, and 15 days of short-day photoperiod treatment was subjected to transcriptome sequencing, and 121.09 G clean data were obtained. The distribution of the proportions of the various reads from the library is given in Table 4. The effective data volume of each sample were distributed in the range of 6.04–7.2G, and the distribution of Q30 bases was 93.7–94.64%, with an average GC content of 44.10%. The data obtained from filtering clean reads were compared with those of the reference genome using HISAT2. The degree of matching between the data of each sample (clean reads) and the reference genome sequence was above 90%, with the highest match at 93.46% and the lowest at 91.88%, indicating that the results obtained by sequencing were accurate and reliable.

**Table 4:** Summary of RNA-seq results.

**Sample**	**Raw reads**	**Clean reads**	**Q30 (%)**	**GC content (%)**	**Total mapped**	**Multiple mapped**	**Uniquely mapped**
CK-5d-1	46.03M	45.11M	93.70	43.39	44,104,797 (97.77%)	1,944,288 (4.31%)	42,160,509 (93.46%)
CK-5d-2	44.66M	43.84M	94.53	42.54	42,569,240 (97.09%)	1,979,914 (4.52%)	40,589,326 (92.58%)
CK-5d-3	47.70M	46.82M	94.64	42.88	45,654,339 (97.50%)	2,196,016 (4.69%)	43,458,323 (92.81%)
SD-5d-1	45.07M	44.28M	94.57	43.03	43,067,558 (97.27%)	1,975,957 (4.46%)	41,091,601 (92.81%)
SD-5d-2	49.65M	48.65M	93.86	43.98	47,584,894 (97.82%)	2,227,964 (4.58%)	45,356,930 (93.24%)
SD-5d-3	42.68M	41.93M	94.39	42.72	40,719,836 (97.11%)	1,845,739 (4.40%)	38,874,097 (92.71%)
CK-10d-1	50.31M	49.31M	94.03	44.33	48,196,864 (97.75%)	2,325,921 (4.72%)	45,870,943 (93.03%)
CK-10d-2	47.65M	46.71M	94.10	44.75	45,724,496 (97.89%)	2,676,489 (5.73%)	43,048,007 (92.16%)
CK-10d-3	47.91M	47.04M	94.16	44.53	46,153,328 (98.11%)	2,590,091 (5.51%)	43,563,237 (92.61%)
SD-10d-1	43.72M	42.89M	94.04	44.79	42,115,641 (98.19%)	2,619,838 (6.11%)	39,495,803 (92.08%)
SD-10d-2	43.37M	42.54M	93.99	44.43	41,766,075 (98.17%)	2,390,006 (5.62%)	39,376,069 (92.56%)
SD-10d-3	50.18M	49.28M	94.24	44.79	48,403,476 (98.22%)	2,844,589 (5.77%)	45,558,887 (92.45%)
CK-15d-1	50.07M	49.09M	94.00	44.58	48,197,147 (98.18%)	2,475,315 (5.04%)	45,721,832 (93.14%)
CK-15d-2	46.03M	45.21M	94.17	44.29	44,392,867 (98.18%)	2,242,198 (4.96%)	42,150,669 (93.22%)
CK-15d-3	47.81M	46.85M	93.97	44.37	45,866,501 (97.90%)	2,196,580 (4.69%)	43,669,921 (93.21%)
SD-15d-1	49.13M	48.36M	94.42	44.99	47,471,908 (98.16%)	3,039,632 (6.29%)	44,432,276 (91.88%)
SD-15d-2	47.47M	46.69M	94.45	44.62	45,812,022 (98.13%)	2,686,977 (5.76%)	43,125,045 (92.37%)
SD-15d-3	48.25M	47.36M	94.13	44.76	46,500,053 (98.19%)	2,949,831 (6.23%)	43,550,222 (91.96%)

### Numerical Analysis of Differentially Expressed Genes (DEGs) in Response to Short-Day Conditions

**Figure 2:**
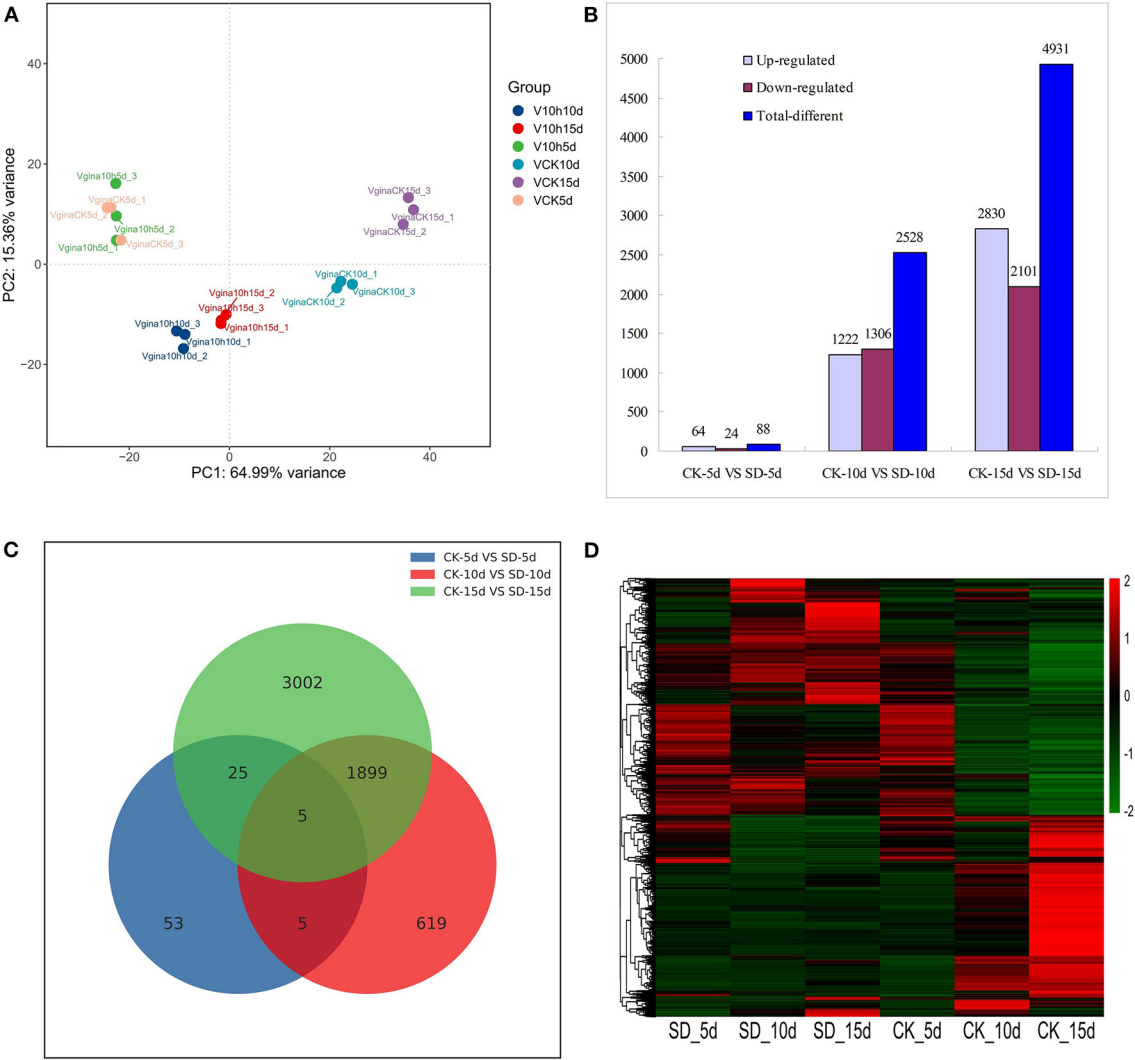
Analysis of differentially expressed genes in response to short-day photoperiod. **(A)** Principal component analysis of the sequenced samples. **(B)** Numbers of the significantly regulated genes under different days short-day photoperiod treatment. The numbers on the horizontal axis reflect different treatment and the vertical axis represents numbers of total, up-and down-regulated genes, respectively. **(C)** Venn diagram analysis of significantly regulated genes under different days short-day photoperiod treatment. **(D)** Heatmap clustering of global pattern of the strongly regulated genes conducted using Hierarchical Clustering (HCL) algorithm under different days short-day photoperiod treatment. The color scale represents the values of lg FPKM (FPKM–Fragments Per Kilobase of transcript per Million fragments mapped.

Principal component analysis (PCA) results showed that the distance among different samples was long; only CK-5d and SD-5d groups were overlapped. For SD-5d, between-group differences in gene expression were not significant, but others showed larger differences. Although the distance was short within different samples, the gene expression similarity within samples was high, and the gene expression of the samples within the group was uniform and consistent (Figure 2A).

The data from the three groups of short-day photoperiod treatments were compared with those from their respective controls, and the results are shown in Figure 2B. The results showed that the total number of differential genes was 7,547, of which 4,116 genes were upregulated and 3,431 genes were downregulated in expression. Among them, 88 differential genes were detected between CK-5d vs. SD-5d, including 64 genes that had upregulated expression and 24 genes that had downregulated expression; 2,528 differential genes were detected between CK-10d vs. SD-10d, including 1,222 genes that had upregulated expression and 1,306 genes that had downregulated expression. The highest number of differential genes was detected between CK-15d vs. SD-15d, with a total of 4,931 differential genes, including 2,830 genes that had upregulated expression and 2,101 genes that had downregulated expression. This indicates that the number of differential genes increased with extended durations of short-day photoperiod conditions and the number of flowering response genes increased after short-day photoperiod inducement. As seen in Figure 2C, the numbers of identical genes in the three groups were 10, 1,904, and 30, respectively, and there were five differential genes in the three groups, with the largest number of differential genes shared between CK-10d vs. SD-10d and CK-15d vs. SD-15d treatments.

The clustering of differential genes can be seen from the heat map in Figure 2D. The gene expression of CK-5d is similar to that of SD-5d, with the lowly expressed genes distributed in the upper and lower parts and the highly expressed genes distributed in the middle. The expression of differential genes changed significantly with prolonged shading time. The highly expressed genes of CK-10d and CK-15d distributed in the lower part and the lowly expressed genes distributed in the upper part. SD-10d and SD-15d treatments showed opposite trends. The aforementioned gene expression results showed that gene expression changed accordingly with prolonged short-day photoperiod treatments, and some genes regulating flowering metabolism such as circadian rhythm, plant hormones, antenna proteins, and photosystem II were upregulated. With different response abilities to short-day photoperiod conditions, some genes in other metabolic pathways were downregulated, indicating that the opposite changing trends of gene expression under CK-10d and SD-10d and under CK-15d and SD-15d were related to the earlier flowering of adzuki bean induced by short day.

### GO Functional Enrichment Analysis of DEGs in Response to Short-Day Photoperiod Conditions

**Figure 3:**
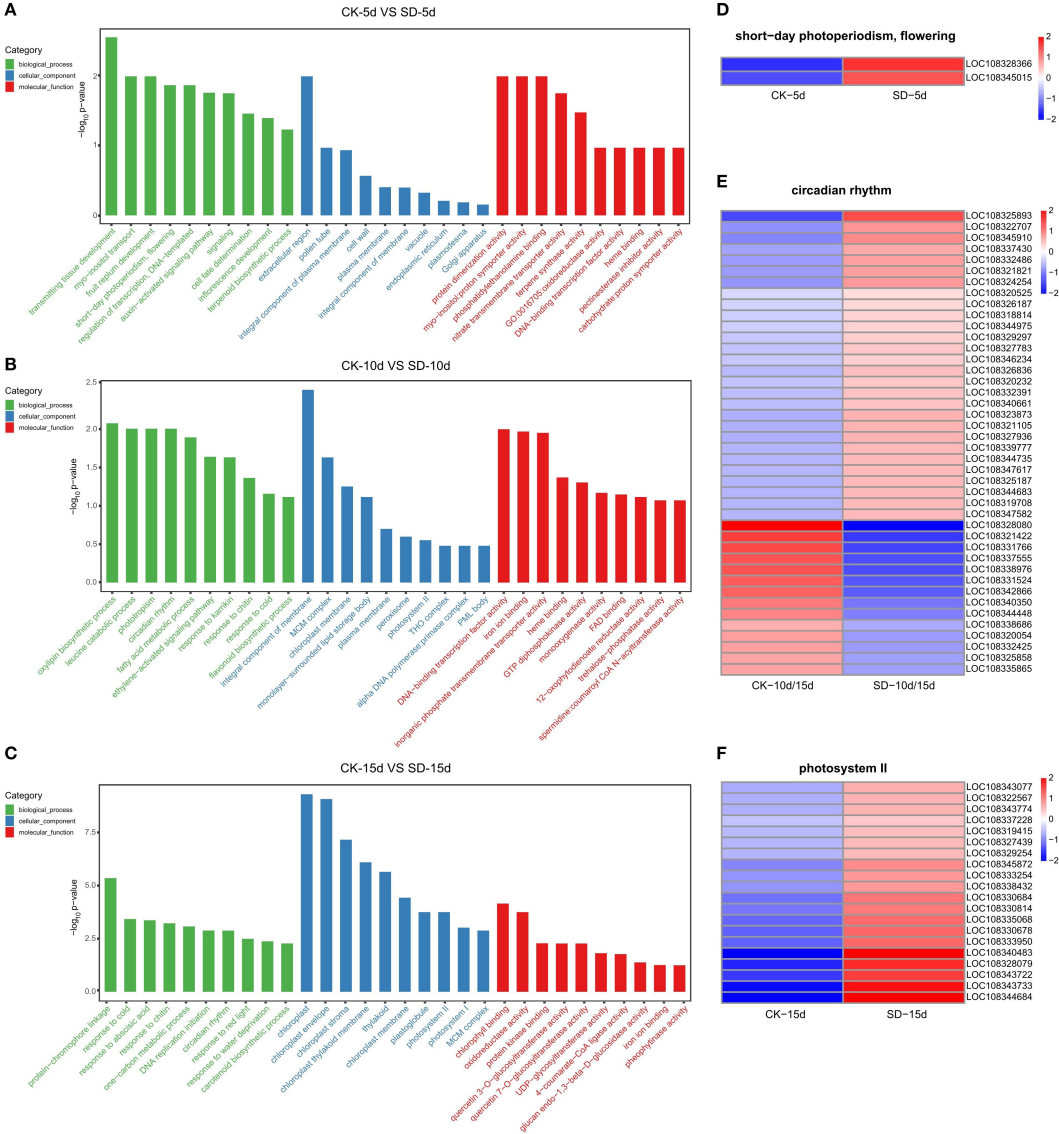
GO enrichment results and heat map clustering analysis of three comparison groups. **(A–C)** GO enrichment between the CK-5d vs. SD-5d, CK-10d vs. SD-10d and CK-15d vs. SD-15d groups, The X coordinates in the diagram are GO entry names, and the Y coordinates are-log10 *p*-values. **(D–F)** Heatmap clustering analysis of short-day photoperiodism, flowering, circadian rhythm and photosystemll functional entries for differential genes. The color scale represents the values of lg FPKM (FPKM–Fragments Per Kilobase of transcript per Million fragments mapped.

GO functional enrichment analysis was performed on three groups of differential genes; 10 GO entries corresponding to a number >2 of the differential genes were each screened for biological processes, cellular components, and molecular functions. The GO functional enrichment results (Figure 3) showed that each group was significantly different and had different light-related functions according to their corresponding −log10 *p*-value in the descending order, among which CK-5d vs. SD-5d differential genes had a functional group related to flowering, which contained two genes that were both upregulated (Figure 3A). CK-10d vs. SD-10d differential genes also had a light-related circadian functional group in the biological processes and contained 42 genes, of which 28 genes were upregulated and 14 genes were downregulated (Figure 3B). CK-15d vs. SD-15d differential genes had significant differential expression, coding for the photosystem II functional group in the cell composition related to light in adzuki bean, which contained 20 genes that were all upregulated. Fron the results of the aforementioned analysis, we could speculate that short-day photoperiod inducement can promote flowering by stimulating the upregulation of flowering genes and adjusting the biological clock by upregulating and downregulating genes in the circadian rhythm, thus controlling the flowering time of adzuki bean. In addition, short-day photoperiod conditions activate key genes in the photosystem to enhance chlorophyll synthesis and provide nutrients for adzuki bean flowering. Under different short-day photoperiod treatment times, entries were enriched by the GO function, and the expression of genes differed.

### Analysis of the KEGG Enrichment Pathway of DEGs in Response to Short-Day Photoperiod Conditions

**Figure 4:**
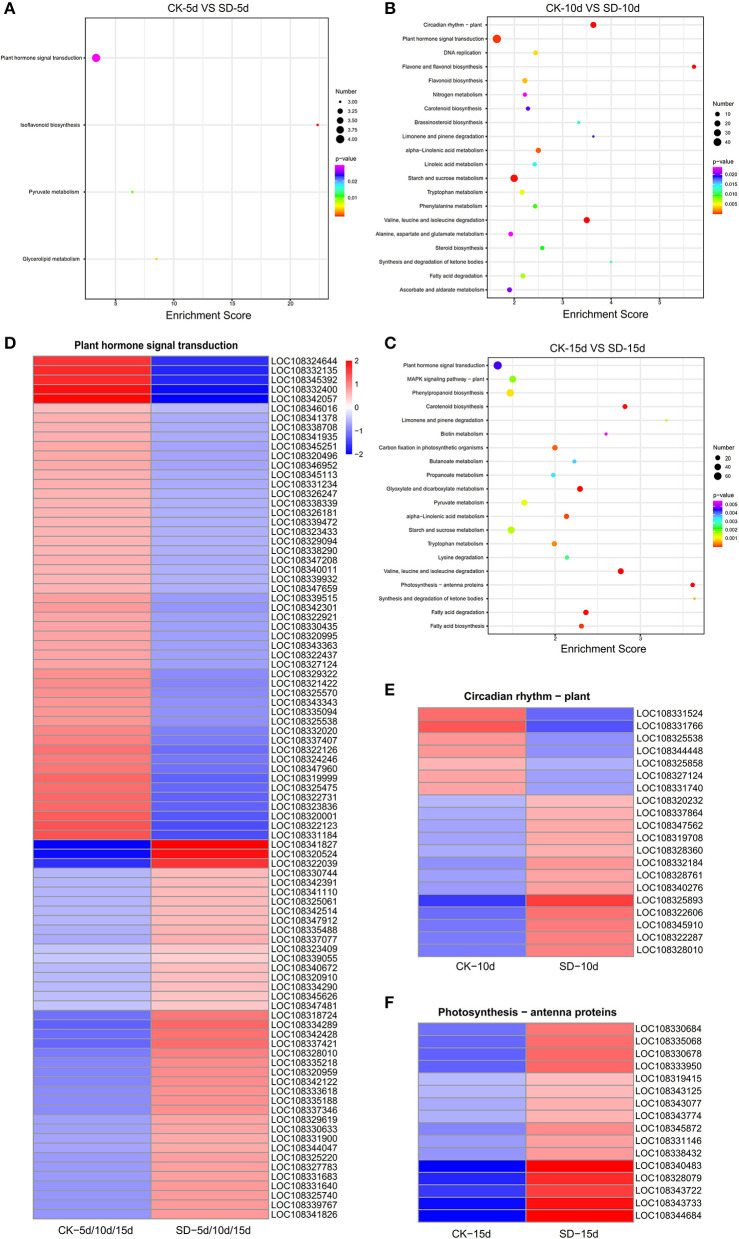
Heat map clustering analysis of KEGG enrichment results of metabolic pathways in the three comparison groups. **(A**–**C)** KEGG enrichment results of metabolic pathways in CK-5d vs. SD-5d, CK-10d vs. SD-10d, CK-15d vs. SD-15d. Notes: the bubble size represents the number of genes that contain the difference. The number of differentially expressed genes in the pathway with large bubbles was larger. The color of the bubbles represents the degree of enrichment, i.e., the pvalue was becoming smaller with the significant degree of enrichment becoming greater ranging from purple to red. **(D–F)** Heat map clustering analysis of differential genes in plant hormone signaling pathways, circadian rhythms and antenna protein metabolism pathways. The color scale represents the values of lg FPKM (FPKM–Fragments Per Kilobase of transcript per Million fragments mapped.

Figure 4 shows the top 20 pathways with the smallest Q values. The number of the DEGs is indicated by the size of the dots, and the different Q ranges are indicated by the color of the dots. The results of KEGG enrichment analysis for each group of the DEGs showed that the hormone signaling metabolic pathway, the circadian rhythm pathway, and the antenna protein pathway were associated with short-day photoperiod conditions, and more genes were enriched in these pathways. CK-5d vs. SD-5d had more genes enriched in the phytohormone signaling metabolic pathway, of which four were upregulated. CK-10d vs. SD-10d had more genes enriched in the phytohormone signaling metabolic pathway and the circadian pathway; 47 genes were enriched in the phytohormone signaling metabolic pathway, of which 19 genes were upregulated and 28 genes were downregulated, and 20 genes were enriched in the circadian pathway, of which 13 genes were upregulated and 7 genes were downregulated. In the bubble plots of KEGG enrichment for CK-15d vs. SD-15d, the phytohormone signaling metabolic pathway and the antenna pathway had the highest number of enriched genes. Out of the 73 genes enriched in the phytohormone signaling metabolic pathway, 31 genes were upregulated and 42 genes were downregulated, and all 16 genes enriched for the antenna pathway were upregulated. It can also be seen that all three groups of DEGs were enriched in the phytohormone signaling metabolic pathway with the highest number of genes, and after combining the three groups of the DEGs, we obtained 91 genes with 40 genes having upregulated expression and 51 genes having downregulated expression. It was shown that the aforementioned DEGs regulated the endogenous hormone levels of adzuki bean by activating or repressing their own expression and thus directly or indirectly regulating the growth and development of adzuki bean under short-day photoperiod conditions. In addition, the circadian pathway and antenna protein pathway were also enriched to a greater extent, indicating that the expression of relevant differential genes in these two pathways was also active under short-day photoperiod inducement.

### GO Function and Expression Analysis of Light-Related Differential Genes in the KEGG Pathway

**Figure 5:**
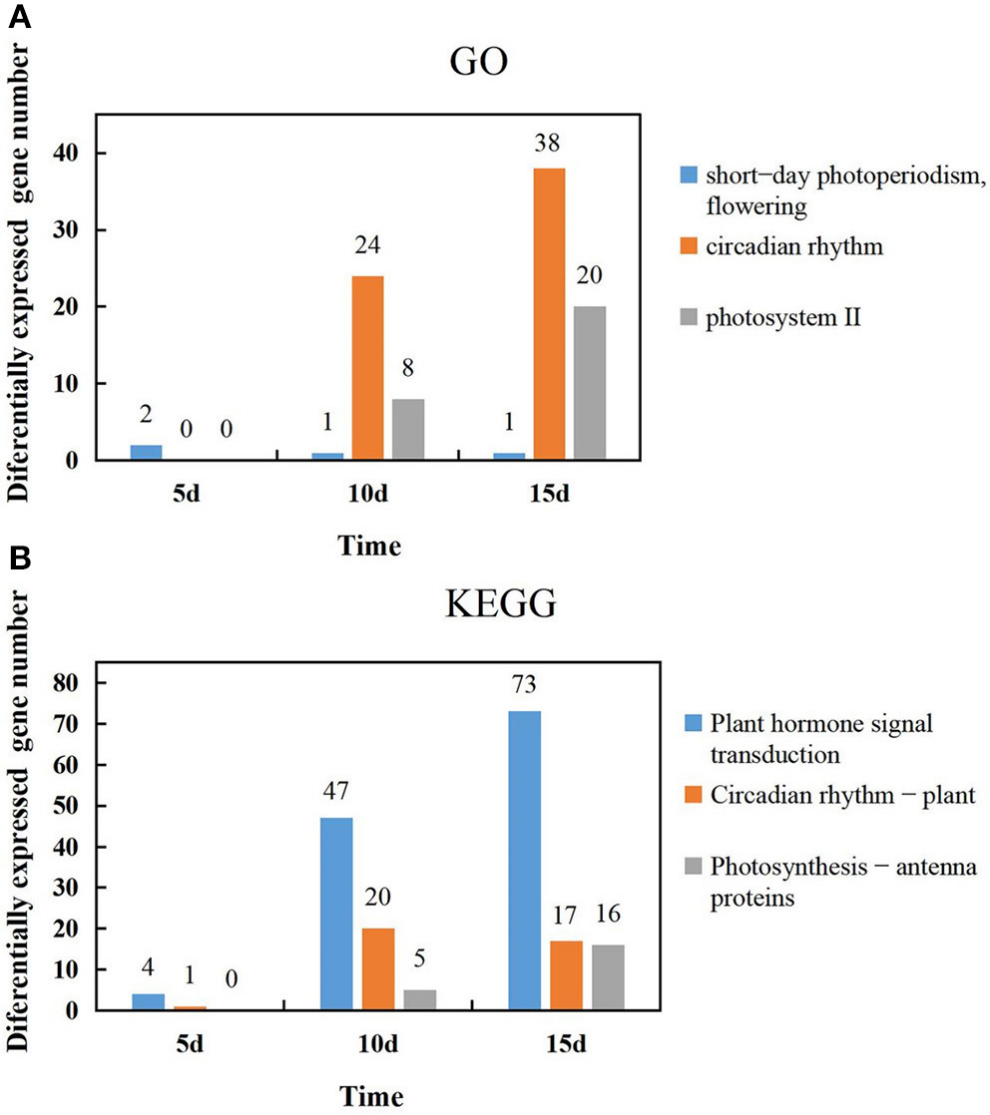
Temporal changes in the number of light-related genes in GO function and the KEGG pathway. **(A)** Changes of the genes number in short-day photoperiodism, flowering, circadian rhythm and photosystem II GO function at 5d, 10d, and 15d. **(B)** Changes of the genes number in Plant hormone signal transduction, Circadian rhythm-plant, Photosynthesis-antenna proteins KEGG pathway at 5d, 10d, and 15d.

The temporal changes in the GO function and the number of differential genes associated with light in the KEGG pathway were analyzed. It was found that most genes showed an increase with extended durations of short-day photoperiod inducement, but a few genes were slightly different; two genes were expressed at 5 days among the GO functional entries of short-day photoperiodism and flowering, while one gene was expressed at both 10 days and 15 days. The number of genes in both circadian rhythm and photosystem II functional entries increased with extended durations of short-day photoperiod inducement, and the variations were 0, 24, 38 and 0, 8, 20, respectively (Figure 5A). This indicates that circadian rhythm and photosystem II functional entries play a dominant role in response to short-day photoperiod inducement. The number of genes in the light-related pathways of plant hormone signal transduction and photosynthesis-antenna proteins increased with extended durations of short-day photoperiod inducement, with variations of 4, 47, 73, 0, 5, and 16, while the number changes of genes in the circadian rhythm pathway showed an increase and then a slight decrease (Figure 5B). Among these three pathways, the largest changes in the number of genes in the phytohormone signal transduction pathway were observed. This indicates that the genes in the metabolic pathway of phytohormone signaling are coordinated with each other to participate in the control of flowering time in adzuki bean after short-day photoperiod inducement.

In each of the phytohormone signaling metabolic, circadian, and antennal protein signaling pathways, 5, 5, and 3 genes, respectively, that were significantly upregulated or downregulated were selected for comparative expression analysis. As few genes were enriched in KEGG in the CK-5d vs. SD-5d comparison group, genes in each pathway were selected in the CK-10d vs. SD-10d and CK-15d vs. SD-15d comparison groups. The genes selected from both the phytohormone signaling metabolic pathway and the circadian pathway were found to be one gene with upregulated expression and four genes with downregulated expression, while genes in the antenna protein signaling pathway mostly had upregulated expression. A total of 13 differentially expressed genes screened were related to light and flowering according to the homolog function (Table 5). It was shown that these genes regulated the growth and development of adzuki bean by activating or repressing their own expression and thus regulating the flowering time of adzuki bean in different metabolic pathways.

**Table 5:** List of different genes associated with light exposure in different signaling pathways.

**KEGG pathway**	**Gene ID**	**gene symbol**	**Regulation**	**q-value** **CK-10dVS SD-10d** **CK-15dVS SD-15d**	**Description**	**Location**	**Functional annotations of orthologs**
Plant hormone signal transduction(KEGG)	LOC108334289	F19P19.31	Up	5.2419E-68 5.1174E-63	Auxin-responsive protein IAA17	Chromosome 5 NC_030641.1	Auxin-responsive protein
	LOC108345251	T20010.80	Down	5.7337E-25 2.9722E-25	Protein transport inhibitor response 1-like	Chromosome 1 NC_030637.1	Leucine-rich repeat, cysteine-containing subtype
	LOC108342301	MJB21.13	Down	1.6981E-17 4.8082E-26	BRI1 kinase inhibitor 1-like	Chromosome 9 NC_030645.1	BRI1 kinase inhibitor 1-like
	LOC108319999	MOE17.8	Down	5.7916E-40 2.5844E-153	Protein ethylene insensitive 3-like	Un NW_016114933.1	Ethylene insensitive 3
	LOC108320001	MOE17.8	Down	1.5701E-35 3.6499E-144	Protein ethylene insensitive 3-like	Un NW_016114933.1	Ethylene insensitive 3
Circadian rhythm-plant (KEGG)	LOC108328360	MKP6.27	Up	1.3414E-18 1.7789E-41	Transcription factor HY5-like	Chromosome 3 NC_030639.1	Basic-leucine zipper domain
	LOC108325858	F19P19.14	Down	2.9319E-16 1.8408E-106	Cryptochrome-1-like	Chromosome 2 NC_030638.1	Cryptochrome/DNA photolyase, FAD-binding domain
	LOC108331524	T3H13.14	Down	1.9432E-53 9.3532E-124	Cryptochrome-1	Chromosome 4 NC_030640.1	Cryptochrome/DNA photolyase, FAD-binding domain
	LOC108344448	F16B22.17	Down	1.4873E-20 1.3516E-97	Putative casein kinase II subunit beta-4	Chromosome 1 NC_030637.1	Casein kinase II subunit beta
	LOC108331766	F17H15.25	Down	1.9738E-62 2.0339E-153	Protein early flowering 3-like	Chromosome 4 NC_030640.1	Protein early flowering 3-like
Photosynthesis-antenna proteins (KEGG)	LOC108328079	F27I1.2	Up	2.3086E-22 1.2264E-122	Chlorophyll a-b binding protein CP29.3, chloroplastic	Chromosome 3 NC_030639.1	Chlorophyll a-b binding protein
	LOC108330684	lhcA-P4	Down/Up	6.1631E-16 1.42167E-33	Chlorophyll a-b binding protein P4, chloroplastic	Chromosome 4 NC_030640.1	Chlorophyll a-b binding protein
	LOC108340483	LHBC1	Up	3.3249E-11 1.8627E-57	Chlorophyll a-b binding protein 13, chloroplastic	Chromosome 8 NC_030644.1	Chlorophyll a-b binding protein

### qRT-PCR Validation of Differential Gene Expression

**Figure 6:**
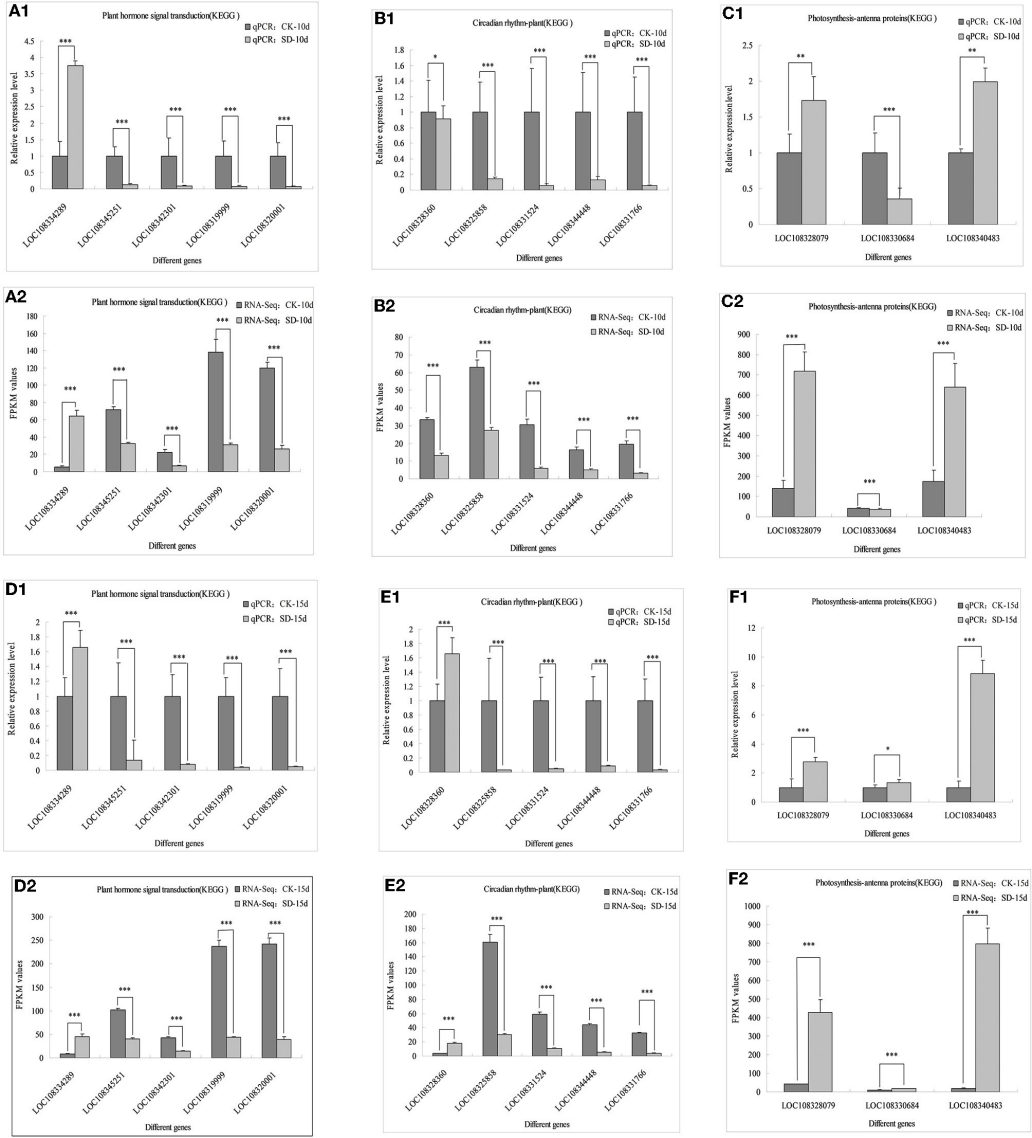
Comparation between qRT-PCR validation and RNA-seq of 13 differentially related photoperiod expressed genes. *Indicates significant difference at 0.05 level, ** and *** indicate extremely significant difference at 0.01 and 0.001 level. **(A1–C1)** The qPCR validation of 13 genes in three metabolic pathways of plant hormone signal transduction, circadian rhythm and photosynthesis-antenna protein were induced for 10 days under 10 h short-day photoperiod inducement; **(A2–C2)** RNA-Seq values of 13 genes in three metabolic pathways of plant hormone signal transduction, circadian rhythm and photosynthesis-antenna protein were induced for 10 days under 10 h short-day photoperiod inducement; **(D1–F1)** The qPCR validation of 13 genes in three metabolic pathways of plant hormone signal transduction, circadian rhythm and photosynthesis-antenna protein were induced for 10 days under 15 h short-day photoperiod inducement; **(D2–F2)** RNA-Seq values of 13 genes in three metabolic pathways of plant hormone signal transduction, circadian rhythm and photosynthesis-antenna protein were induced for 10 days under 15 h short-day photoperiod inducement.

To verify the reliability of adzuki bean transcriptome data, 13 genes with significant differences in the plant hormone, circadian rhythm, and antenna protein pathways were verified by qRT-PCR again with new adzuki bean samples using the same treatment. The expression of five genes in the metabolic pathway of phytohormone signaling differed, where expression of *LOC108334289* significantly increased under SD-10d and SD-15d treatments compared with that in the control, whereas *LOC108345251, LOC108342301, LOC108319999*, and *LOC108320001* had significantly lower expression than those in the control (Figures 6A1,D1). In the circadian rhythm pathway, *LOC108328360* genes had significantly lower expression under SD-10d treatment, but significantly increased expression under SD-15d treatment. Moreover, *LOC108325858, LOC108331524, LOC108344448*, and *LOC108331766* had significantly lower expression under both SD-10d and SD-15d treatments than in the control (Figures 6B1,E1). In the antenna signaling pathway, *LOC108330684* had significantly reduced expression compared with that in the control under SD-10d treatment but significantly increased expression under SD-15d treatment, with *LOC108328079* and *LOC108340483* having significantly elevated expression compared with the control under SD-10d and SD-15d treatments (Figures 6C1,F1). In addition, qRT-PCR analysis on detecting expression patterns of 13 genes indicated that they were completely consistent with the transcriptional sequencing results RNA-Seq values (Figures 6A1A2–F1F2), and the differences of 13 genes in different signaling pathways reached a significant level. Only *LOC108328360, LOC108328079*, and *LOC108340483* genes had different differences under 10-day treatment, while LOC108330684 genes had different differences under 15-day treatment. These results indicated that these transcriptome sequencing data were accurate and reliable.

## Discussion

Adzuki bean (*Vigna angularis*) originated from and produced in China, with cropland area and total production as one of the largest in the world. Adzuki bean can be adapted to a wide range of uses and is a grain legume of economic importance. As a model crop with a short growth cycle and small genome, adzuki bean is often used to study model plants through transcriptomics to reveal the biological mechanisms of different genes and has important applications in the genetic improvement of legume crops (Yamada et al., 2001). Currently, transcriptome sequencing technology is widely used in the discovery of new genes and comparative genomics studies (Wang et al., 2014; Prince et al., 2015; Liu et al., 2017; Zhang et al., 2017). In this study, we used ‘Tang shan hong xiao dou', which is sensitive to short-day photoperiod conditions, as the material to construct a cDNA library of bean under full-day and short-day photoperiod treatment conditions, and we conducted transcriptome sequencing using Illumina sequencing technology to screen DEGs. DEGs increased with extended durations of short-day induction. Short-day photoperiod inducement of 15 days had the highest number of 4,931 DEGs, of which 2,830 were upregulated and 2,101 were downregulated. In terms of GO functional enrichment of DEGs, in CK-5d vs. SD-5d comparison, the differential genes associated with short-day photoperiod conditions were mostly focused on the biological process of flowering promotion, but there were only two genes for this function and related to flowering time; both are described as protein HEADING DATE 3B-like (Hd3) and protein FLOWERING LOCUS T-like (FT), respectively. Related findings in other species showed that Hd3 is a homolog of FT that can exert its floral function as an anthocyanin gene, with the protein expressed in leaf vascular bundles and transferred to the apical meristem through the vascular bundle to promote plant flowering (Li et al., 2014). It was speculated that adzuki bean was blossomed earlier after 5 days of short-day photoperiod inducement, being related to accumulation and transportation of florigen. Through the biological processes, the comparison of CK-10d vs. SD-10d was dominated by 42 differentially expressed circadian rhythm-related genes, of which 28 genes were upregulated and 14 genes were downregulated. Previous studies on other crops showed that the GI-CO-FT cascade in circadian pathways acts as a regulator in determining the flowering behavior (Nakamichi et al., 2020; Liu et al., 2021; Wang et al., 2021). It was speculated that adzuki bean may also participate in this pathway to promote flowering under short-day photoperiod inducement. The CK-15d vs. SD-15d comparison showed that there were 20 DEGs of PS II in the cellular group, and all these genes reached significant levels and were upregulated. It is indicated that these genes may be related to the antenna protein genes. Peripheral light-capturing antenna of photoperiod system II can capture and transmit light energy, improve the utilization rate of light energy, and promote early flowering of adzuki bean under short-day photoperiod inducement conditions. By comparing GO temporal changes in the number of genes in different functions, the number of genes directly promoting flowering in adzuki bean through short-day photoperiod inducement did not increase with the duration of short-day photoperiod condition treatments but rather decreased. However, PS II and circadian rhythm function-related genes increased with the extension of the short-day photoperiod inducement duration, indicating that the gene related to the function of photoperiod-induced plant flowering did not play a dominant role, while PS II and circadian rhythm function might be the key genes regulating the growth and development of adzuki bean under short-day photoperiod inducement.

The results of the KEGG metabolic pathway analysis revealed that the metabolic pathway of phytohormone signaling associated with short-day photoperiod inducement was enriched with the most DEGs, followed by the circadian pathway, and the antenna protein pathway. This suggests that these three pathways may be critical for short-day photoperiod inducement to interfere with the growth and development of adzuki bean and that these genes may be key factors or candidate genes affecting the regulation of short-day photoperiod inducement in adzuki bean seedlings. This is similar to the findings obtained in previous studies using transcriptome sequencing in other crops under short-day photoperiod inducement (He et al., 2017; Xiao et al., 2017; Gao et al., 2021). In addition, we selected 5, 5, and 3 candidate genes with significant differences in regulating flowering through the phytohormone, circadian, and antenna protein metabolism pathways, respectively, because adzuki bean is most closely associated with the phytohormone signaling pathways under short-day photoperiod inducement, and the phytohormone signaling pathways play an important role in the induction of flower formation in plants. By transcriptome analysis, 5 candidate genes in the phytohormone metabolic pathway was described as auxin-responsive protein IAA17, transport inhibitor response 1-like protein, BRI1 kinase inhibitor 1-like protein, and ethylene insensitive 3-like protein. Related findings in other species showed that auxin (IAA) promotes female flower development by inducing ethylene synthesis (Song S. S. et al., 2013); transcription of TIR1 is induced by IAA and ABA; auxin can enhance the interaction between TIR1 and (Aux/IAA) (Yun et al., 2011); the BRI1 kinase repressor can inhibit BRI1 by acting in conjunction with other receptor proteins, eliciting BRI1 in an activated state and prompting brassinolactone gene expression to regulate the flowering time in plants (Jia et al., 2020); and the nuclear protein EIN3 binds to the ethylene reaction gene *ERF1* to initiate a series of transcriptional cascades regulating the expression of ethylene target genes (Kosugi and Ohashi, 2000). In this study, short-day photoperiod conditions not only promoted floral bud differentiation in adzuki bean but also advanced flowering time and flowering promotion rate. The analysis of the transcriptome data revealed that the largest numbers of DEGs associated with phytohormone signaling were mainly growth hormone, gibberellin, and ethylene (ETH), suggesting that short-day photoperiod inducement of flowering in adzuki bean was due to the synergistic effect of multiple phytohormones, among which IAA may play a dominant role. This has some similarities with previous studies (Yang et al., 2014; Zhang et al., 2015). These results suggest that the flowering time of adzuki bean may be related to the change in endogenous hormone levels caused by the upregulation/downregulation of DEGs.

Transcriptome analysis revealed that 5 candidate genes involving the circadian rhythm were described as protein early flowering 3-like, cryptochrome-1-like, cryptochrome-1, putative casein kinase II subunit beta-4, and transcription factor HY5-like, respectively. In other species, we found that HY5 indirectly regulates the synthesis and accumulation of anthocyanins in plants by activating the transcription of itself and downstream target genes after receiving signals transmitted by upstream photoreceptors (Zhang et al., 2010). CRY can act with PHY and PHOT proteins to regulate COP1, a downstream factor in the photoreceptor pathway, to modulate the flowering time in plants (Li et al., 2018). Casein kinase 2 (CK2) is involved in many important physiological processes, such as physiological clock, photoperiod, and flower development of plants (Jayant and Enamul, 2012). EFL3 plays an important role in regulating plant early flowering through the circadian clock pathway (Hicks et al., 2001). It has been suggested that the circadian clock system can coordinate external light temperature signals and the endogenous metabolic and developmental state cues, outputting circadian diurnal signals to regulate plant growth and development after short-day inducement; 3 antenna protein candidate genes were descripted as chlorophyll a-b-binding proteins CP29.3, P4, and 13, chloroplastic, respectively. All these genes in *Barley* and *Arabidopsis* have main functions in the capturing and transmitting of light energy, balancing the excitation energy of PS I and PS II by capturing and transmitting light energy in the photosystem and especially in maintaining the structure of the vesicle-like membrane and responding to external environmental changes and photoprotection (Xia Y. S. et al., 2012; Xu et al., 2012). In this study, 16 genes were enriched by KEGG to the antenna protein, including five LHCII-encoded major photopigment protein complexes, all of which were upregulated in expression and associated with photoperiodic responses, suggesting a stress response of adzuki bean after a short-day photoperiod inducement to improve its own light energy utilization to accumulate more compounds to maintain the cellular osmotic potential. These findings were verified in rice, *Arabidopsis* (Pavan, 2010), *Ginkgo* (Wang et al., 2015), and *Mongolian wheatgrass* (Zhao et al., 2017). By analyzing the temporal changes in the number of genes of different metabolic pathways in KEGG, we found that the number of genes in circadian rhythm genes showed a trend of increase and then decrease in the three groups of CK-5d vs. SD-5d, CK-10d vs. SD-10d, and CK-15d vs. SD-15d, while the number of genes in the phytohormone signal transduction metabolic pathway increased with the extension of short daylight, with the largest number of genes in the phytohormone signal transduction metabolic pathway. Thus, the hypothesis is that, after short-day photoperiod inducement, the genes of the phytohormone signal transduction metabolic pathway play a dominant role in growth and development, and flowering is regulated through the synergistic effect of multiple phytohormones. The results of these studies elucidate the molecular regulatory basis of photoperiod-induced flowering in adzuki bean and provide a theoretical basis for photoperiod-adapted breeding of adzuki bean germplasm, as well as lay the foundation for functional validation of DEGs for future research.

## Conclusion

Comparing the transcriptomes of CK-5d vs. SD-5d, CK-10d vs. SD-10d, and CK-15d vs. SD-15d in the late maturing variety “Tang shan hong xiao dou” indicated that there were 88, 2,528, and 4,931 DEGs, respectively, indicating that the DEGs increased with the extension in the duration of short-day photoperiod inducement. The 15d short-day photoperiod condition was observed to have the greatest effect on flower bud differentiation and the flowering time of adzuki bean. To reveal the expression of genes related to short-day photoperiod inducement and flowering, the analysis of GO and KEGG revealed that the DEGs were mainly associated with functional entries in flowering, photosystem, and circadian rhythm. Pathway analysis of differential protein-encoding genes using the KEGG database further revealed that most genes were mainly enriched in the hormone signaling metabolism, circadian rhythm, and antenna protein pathways; 13 flowering-associated candidate genes were identified in the three pathways, which may be involved in the regulation of flowering time in adzuki bean. The present study provides insights into the elucidation of the gene transcription and regulatory network of flowering time and an empirical basis for further studies of the development and flowering characteristics in adzuki bean species.

## Data Availability Statement

The datasets presented in this study can be found in online repositories. The names of the repository/repositories and accession number(s) can be found below: NCBI - PRJNA817421.

## Author Contributions

WD and YZ designed the experiments and revised the manuscript. DL and LZ performed the field experiment and collected samples. LZ and BY collected phenotypic data in Baoding in 2021. WD and DL performed data analysis and wrote the manuscript. All authors read and approved the final manuscript version.

## Funding

This work was supported by the Hebei Province Natural Science Foundation for Youth (C2021204405), the China Agriculture Research System of MOF and MARA – Food Legumes (CARS-08-G-22), and the National Key Research and Development Program of China (2021YFD1901004-2).

## Conflict of Interest

The authors declare that the research was conducted in the absence of any commercial or financial relationships that could be construed as a potential conflict of interest.

## Publisher's Note

All claims expressed in this article are solely those of the authors and do not necessarily represent those of their affiliated organizations, or those of the publisher, the editors and the reviewers. Any product that may be evaluated in this article, or claim that may be made by its manufacturer, is not guaranteed or endorsed by the publisher.
